# Metformin: Focus on Melanoma

**DOI:** 10.3389/fendo.2018.00472

**Published:** 2018-08-21

**Authors:** Emilie Jaune, Stéphane Rocchi

**Affiliations:** ^1^INSERM U1065, Centre Méditerranéen de Médecine Moléculaire (C3M), Nice, France; ^2^Université de Nice Sophia Antipolis, UFR de Médecine, Nice, France

**Keywords:** biguanides, metformin, melanoma skin cancer, cancer treatment, AMPK pathway

## Abstract

Metformin is the most common biguanide used in the treatment of diabetes, with 120 million treated patients worldwide. Metformin decreases hyperglycemia without inducing hypoglycemia in diabetic patients and is very well tolerated. The principal effects of metformin are to decrease hepatic gluconeogenesis and increase glucose absorption by skeletal muscles. These effects are primarily due to metformin's action on mitochondria, which requires the activation of metabolic checkpoint AMP-activated protein kinase (AMPK). AMPK is implicated in several pathways, and following metformin activation, it decreases protein synthesis and cell proliferation. Many studies have examined the role of metformin in the regulation of cancer cells, particularly its effects on cancer cell proliferation and cell death. Encouraging results have been obtained in different types of cancers, including prostate, breast, lung, and skin cancers (melanoma). Furthermore, many retrospective epidemiological studies in diabetes patients have shown that metformin treatment decreased the risk of cancers compared with other antidiabetic treatments. In this review, we will discuss the effects of metformin on melanoma cells. Together, our novel data demonstrate the importance of developing metformin and new biguanide-derived compounds as potential treatments against a number of different cancers, particularly melanoma.

## Introduction

Biguanides are molecules derived from guanidine and are used in diabetes treatment. Guanidine is extracted from *Galega officinalis*, a plant used in medicine for many years. Indeed, this plant was used for its antidiabetic properties before its effects on glycaemia were discovered in the 1920s (1). Since this time, many guanidine-derived compounds have been used in type 2 diabetes, such as buformin, phenformin, and metformin. At first, metformin was not truly compared with other guanidine-derived compounds because of its less important effects on insulin sensitivity. Other biguanides, phenformin, and buformin were widely used in diabetic treatments starting in 1920 until their high toxicity in patients was discovered in 1930 (2). Afterwards, biguanides were no longer used in type 2 diabetes treatment until a study by French chemist Jean Sterne in 1957, where he showed metformin's effects on type 2 diabetes without apparent toxicity (3). Thanks to this study, metformin received marketing authorization in Europe in 1958 and in the USA in 1995 (2). Currently, metformin is the most prescribed antidiabetic medication in the world, and it is used to treat more than 120 million people (4).

After years of treatment with metformin, retrospectives studies showed that diabetes patients had decreased cancer incidence with metformin compared to treatment with another antidiabetic drug (5). Afterwards, studies confirmed these results (6, 7), and many groups focused their research on metformin's effects on cancer cells. In this study, we will particularly focus on metformin's effects on melanoma.

Cutaneous melanoma is a malignant cancer that rises from the transformation of melanocytes. These cells are normally responsible for the synthesis of melanin, which is a photoprotector pigment. Melanoma is widespread with 200,000 new cases every year and 65,000 melanoma-associated deaths. Its incidence doubles every 10 years, and although it represents only 10% of all skin cancers, melanoma is responsible for 80% of skin cancer deaths, which constitutes a real public health problem (8). Melanoma is the most aggressive skin cancer and possesses a strong invasive capability that enables the development of metastasis principally in the lymph node, liver, lung but also in the central nervous system. Metastatic melanoma is one of the deadliest cancers because of the inefficacy of current therapies.

For 15 years, targeted therapy against BRAF(mutated in 50–60% of primary melanoma) or MEK protein has been developed, and some of these treatments have been commercialized, including BRAF inhibitors, such as vemurafenib (or PLX4032) and dabrafenib, and MEK inhibitors, such as cobimetinib, or trametinib (9). The first results with these therapies seem promising with an increase in overall survival and shrinkage of the primary tumor. However, after few weeks of treatment, patients develop a strong resistance to these therapies, enabling metastatic growth and relapse (10, 11). Furthermore, melanoma cells have the ability to escape the immune response. Due to this observation, current therapeutic approaches try to allow immune system activation to kill melanoma cells (12, 13). Currently, two different antibodies are commercialized: ipilimumab (anti-CTLA-4) and nivolumab (anti-PD-1). ipilimumab targets CD4+ T cells, whereby its inhibition allows T-cell activation. This treatment increases patient survival rate, but only 15–20% of patients respond to this treatment (14). PD-1 is also expressed on T cells, and its expression inhibits T-cell activation. Its target, PDL-1, is widely found in melanoma cells. PD-1 treatment shows a response in ~30–40% of patients (15). Even if these responses result in an objective and long-lasting response, ~55–60% of patients do not respond to these treatments. The identification of new antimelanoma compounds is essential for developing new therapies.

## Principal effects of metformin in type 2 diabetes treatments

In type 2 diabetes patients, metformin (N,N-dimethylbiguanide) exerts its antidiabetic function by decreasing the insulin resistance of glucose-intolerant patients and hepatic gluconeogenesis. Indeed, the liver is considered to be the principal site of action of metformin, where it can act on gluconeogenesis, glycolysis, and glycogen synthesis. In type 2 diabetes patients, hepatic gluconeogenesis is increased relative to healthy patients. However, under metformin treatment, glucose absorption and general levels of glucose can decrease to 75% (16). Furthermore, this molecule also increases the high absorption of glucose by skeletal muscles, which improves its effects on glucose homeostasis. In general, metformin increases glucose absorption by increasing the plasma membrane translocation of glucose receptors, such as glucose transporter 1 (GLUT-1), in both hepatic cells (17) and skeletal muscle cells (18). In addition, this compound highly increases the expression of insulin receptor substrate 1 and 2 (IRS-1 and 2), which enhances glucose absorption.

Interestingly, metformin also blocks the effects of glucagon, which normally enhances gluconeogenesis, by inhibiting essential enzymes in this process and stimulating glycolysis via the alteration of numerous enzymes in this signaling pathway (19).

However, we currently do not understand all the mechanisms of actions of metformin in these patients. Interestingly, a recent study showed the effect of metformin on the intestinal microbiota and its impact on metabolism in obese mice (20). Indeed, type 2 diabetes seems to be impacted by the intestinal microbiota (21), and therefore, the effects on the microbiota could be partly responsible for metformin's effects in type 2 diabetes patients.

## Metformin acts as an anticancer agent

### Retrospective studies

Diabetic patients possess a higher risk of developing cancers than healthy patients, which is partly due to increasing levels of circulating growth factors, such as insulin or insulin growth factor 1 and 2 (IGF-1 and 2) (22). In this context, many retrospective analyses in type 2 diabetes patients have shown that metformin possesses antitumoral proprieties (5–7). In *Evans* et al. diabetic patients treated with metformin presented less cancer than patients treated with other antidiabetics. Following this study, many investigations have shown the antineoplastic effects of metformin in numerous cancer types (6, 23–25). For example, a study compared the effects of three different treatments, metformin, insulin, or sulfonylureas, over 5 years in ~10,300 diabetes patients. The results showed that patients treated with metformin have a lower cancer-related mortality rate than patients treated with other treatments (23). Inversely, the study by Currie et al. showed that patients treated with insulin developed more solid cancer than those treated with metformin (25). Another study observed that 7.3% of type 2 diabetes patients treated with metformin developed cancers compared with 11.6% of patients treated with other antidiabetics (6). In a more specific retrospective study, it was shown that the use of metformin for long-term treatment in men decreased prostate cancer development by 34% compared to patients treated with other antidiabetic drugs (26). In women, metformin treatment induced a 56% decrease in the breast cancer risk of diabetic patients (24). Recently, a study in a Korean population with type 2 diabetes showed a decrease in cancer development for patients treated with long-term metformin (5.8 years) with an incidence of 13.2 per 1000 compared with an incidence of 21.8 per 1000 in patients with another treatments (27).

In 2010, a short-term clinical study (1 month) performed in non-diabetic patients showed the significant effect of metformin on the development of rectal aberrant crypt foci (precancerous lesions) and the proliferation of colonic epithelial cells (28). Currently, 304 clinical trials have been registered on metformin treatment in different cancer types (ClinicalTrial.gov; March 2018).

### Mechanisms of action of metformin on cancer cells

Consequently, many laboratories have tried to understand the mechanisms of action of metformin in different types of cancers, such as lung, prostate, and ovarian cancers or melanoma. The *in vitro* effects of metformin, alone or in combination with other drugs, have been studied in many different cancers (29–32). Moreover, numerous *in vivo* studies have demonstrated the efficacy of metformin in decreasing tumoral growth (33, 34).

#### Indirect effects of metformin

**Figure 1:**
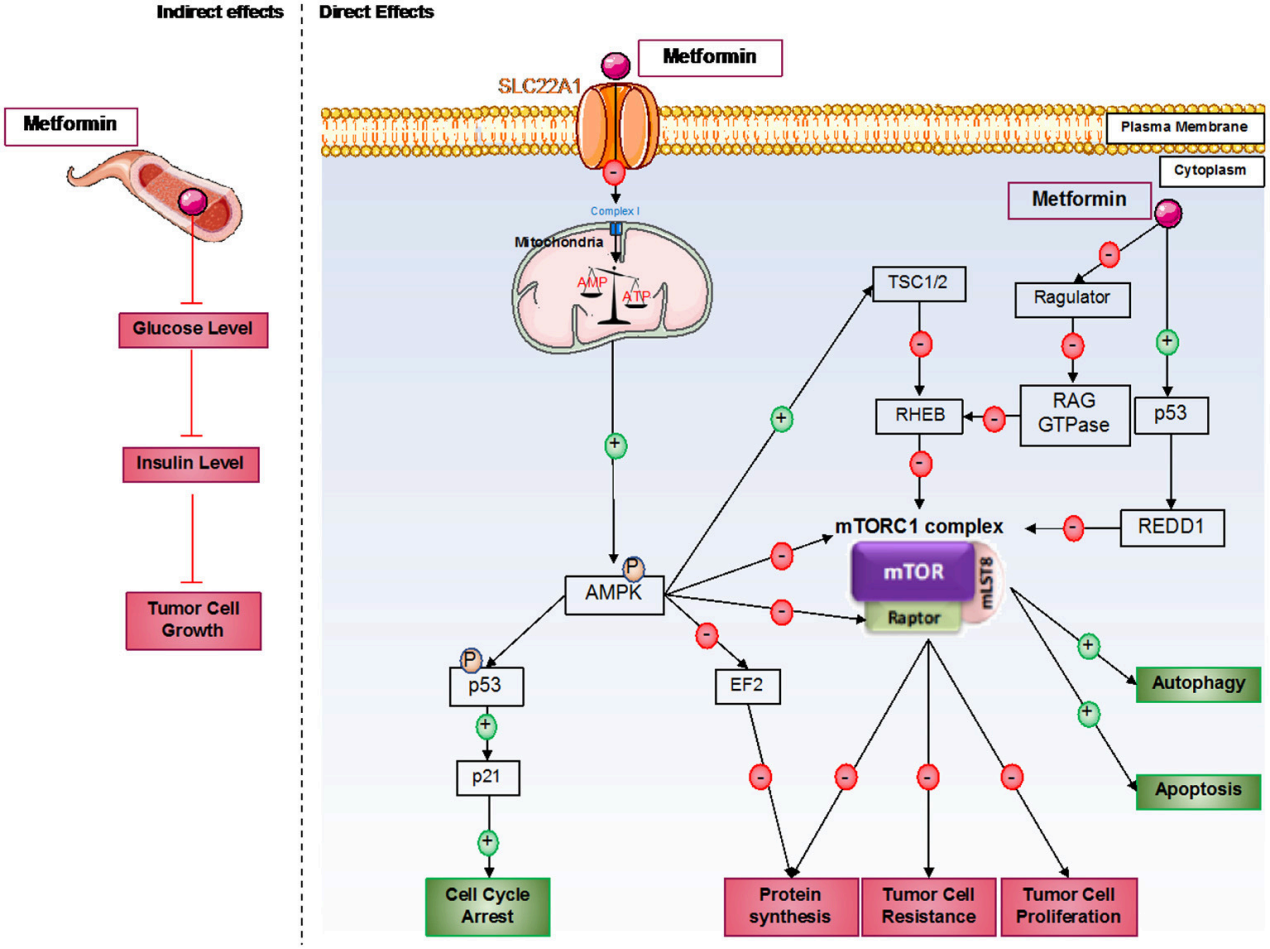
Direct and indirect mechanisms of action of metformin in cancer cells. Metformin induces antitumorigenic effects by both indirect and direct mechanisms. In the blood circulation, metformin decreases glucose levels and therefore insulin levels; insulin can act as a growth factor in tumor cells. For direct effects, metformin acts by AMPK-dependent and independent effects. Generally, metformin induces inhibition of the mTORC1 pathway, which involves an essential protein complex in cellular processes, including protein synthesis and cell proliferation; this complex also promotes tumor cell resistance to therapies. Furthermore, metformin induces cell cycle arrest by p53 activation.

In these studies, different mechanisms have been identified to explain metformin's effects on cancer cells. The first mechanism is an indirect effect of metformin. Indeed, in different cancers, such as breast, colon, or prostate cancer, hyperinsulinemia and obesity induced by insulin and IGF1/2 are associated with poor prognosis (35). Interestingly, metformin decreases circulating insulin levels in patients. Indeed, the transcription of key genes inhibits gluconeogenesis by metformin in the liver, and increased glucose absorption in skeletal muscle cells involves a decrease in blood glucose levels, decreasing circulating insulin levels (36). Therefore, metformin decreases tumoral growth by its inhibition of circulating insulin levels (Figure 1). Furthermore, in a mouse model, metformin inhibited lung cancer cell growth induced by hyperinsulinemia and obesity by decreasing the circulating level of insulin and by activating the AMPK pathway (37). Finally, in non-diabetic woman with breast cancer, a study showed that metformin decreased circulating insulin levels by 22% and increased insulin sensitivity by 25% (38). These results confirm that a decrease in insulin induced by metformin can be considered a new potential mechanism in metformin inhibition of tumorigenesis. As we described previously, metformin seems to impact the microbiota in type 2 diabetes patients (20). Therefore, it will be interesting to study the impact of metformin on the microbiota in different cancer types.

#### Direct effects of metformin

However, the principal effects of metformin on cancer cells are direct effects, which predominantly induce mammalian target of rapamycin complex 1 (mTORC1) inhibition (Figure 1). mTORC1 is a protein complex composed of five different proteins: DEP domain-containing mTOR interacting protein (DEPTOR), mammalian LST8/G-protein β-subunit like protein (mLST8), regulatory-associated protein of mTOR (RAPTOR), proline-rich AKT substrate of 40 kDa (PRAS40), and mammalian target of rapamycin (mTOR). This complex is implicated in many cellular processes but principally in protein synthesis regulation, which is essential for cellular growth. This complex is often activated in cancer cells and can be associated with cancer therapy resistance. Furthermore, mTORC1 plays a critical role in the proliferation and growth of normal stem cells and cancer stem cells. mTOR's implication in cancer stem cell proliferation has been demonstrated in various cancer types, such as colon, pancreas, or breast cancer (39–41).

Depending on the cancer type, many different mechanisms have been discovered to explain the inhibition of mTORC1. The principal one induces AMPK pathway activation after mitochondria dysregulation by metformin. Indeed, at the cellular level, metformin principally acts on mitochondria by inhibiting complex I of the mitochondrial respiratory chain, which disrupts ATP production in the cell (42) and induces AMPK activation (43). A recent study also showed that metformin dysregulates mitochondrial functions *via* calcium flux release (44). Indeed, metformin induces endoplasmic reticulum (ER) stress, which releases calcium into the cytoplasm of the cell. This calcium release induces higher calcium absorption by the mitochondria, which results in mitochondrial swelling. AMPK is an energy sensor that plays an important role in many metabolic pathways involved in restoring energetic balance within the cell (45). In addition, when AMPK activation is sustained, it can play an important role in different cellular processes, such as cell growth and proliferation, cell cycle regulation, cell polarity, apoptosis, and autophagy (46). After metformin induction, AMPK seems to be activated in cancer cells on threonine 172 by liver kinase B1 (LKB1) (47). LKB1 is deleted in many different tumors, such as tumors in cervical or lung cancer, showing the link between LKB1 expression and cancer predisposition. The LKB1/AMPK pathway inhibits mTOR expression *via* the activation of tuberculous sclerosis complex 1 and 2 (TSC1 and TSC2), which induces the dysregulation of protein synthesis, thereby inhibiting tumoral cell proliferation. Interestingly, AMPK can also directly inhibit RAPTOR, a positive regulator of mTOR (48).

Furthermore, metformin can inhibit mTORC1 by AMPK-independent effects (49). Some of these effects are due to mTORC1 inhibition *via* recombinant activating gene (RAG) GTPase family protein inhibition (50). Indeed, RAG GTPases recruit mTORC1 *via* RAPTOR interactions at lysosomal surfaces, where they are activated by Ras homolog enriched in brain (RHEB). Metformin can also directly inhibit Ragulator (51). In prostate cancer, it has been shown that metformin can induce cancer cell death by p53/regulation in development and DNA damage responses 1 (REDD1) pathway activation, which induces the inhibition of mTOR, thereby inhibiting tumor growth (52).

Metformin inhibits cancer cell proliferation by mTORC1-independent mechanisms. Indeed, AMPK can directly phosphorylate p53 on serine 15, which increases p21 expression and enhances cell cycle arrest (53). It has also been described that metformin-induced cell cycle arrest is mediated by cyclin D1 inhibition and Rb dephosphorylation in prostate cancer cells (29) or by an AMPK-dependent mechanism requiring the downregulation of cyclin D1 and implication of p21 and p27 in breast cancer cells (54). A new mechanism implicating the upregulation of micro-RNA34a in renal cancer cells has been described to induce G0-G1 cell cycle arrest under metformin treatment (55). Furthermore, metformin-induced G1-cell cycle arrest has also been observed in pancreatic, glioma, endometrial, and ovarian cancer cells (56). Recent studies in glioblastoma and ovarian cancer cells have also shown cell cycle arrest in G2/M induced by metformin (57, 58). In addition, metformin can induce cell cycle arrest in the S phase in triple-negative breast cancer (54). Metformin inhibits different genes implicated in cell division, such as genes encoding tubulin or histones, which enhances cell cycle arrest (56). AMPK also inhibits protein synthesis *via the* inhibition of elongation factor 2 (EF2) protein (59). Furthermore, under AICAR stimulation, active AMPK can decrease fatty acid synthase (FAS) expression in prostate cancer cells (60). This enzyme is essential for fatty acid synthesis, which is also essential for cell proliferation.

Finally, the inhibition of mTORC1 also induces cell death mechanism activation, which induces cancer cell death. For the autophagy process, mTORC1 inhibits the initial step of phagophore formation. This complex also inhibits unc-51 like autophagy activating kinase 1 (ULK1), an essential kinase for autophagy induction (61). Activated AMPK induced by metformin enhances autophagy initiation *via* inhibition of the mTORC1 complex by phosphorylation of TSC2 on serine 1345 (62, 63). AMPK directly phosphorylates ULK1 and induces mTOR-independent autophagy (61). For the apoptosis process in cancer cells, it has been shown that autophagy induction enhances caspase-dependent apoptosis (64). In adipocytes and under AICAR stimulation, AMPK activates apoptosis processes *via* eukaryotic initiation factor 2 α (eIF2α) regulation (65). Moreover, activation of AMPK stimulates the phosphorylation of p53 on serine 46, which is essential in apoptotic type I programmed cell death induction (66). Different studies in the pancreas and in endometrial cancers showed that the antitumor effects of metformin involve the induction of AMPK-dependent apoptosis (67). Finally, the LKB1/AMPK pathway activated by nutrient deprivation increases cyclin-dependent kinase inhibitor 1B (p27), which enhances autophagy and apoptosis processes in cancer cells (68).

In addition, AMPK activation by metformin induces many different antitumor effects *via* the inhibition of c-MYC or hypoxia-inducible factor-1 α (HIF-1α) (69). Metformin activates the DNA damage reparation pathway *via* ataxia telangiectasia mutated (ATM) activation, which inhibits tumor growth (70).

In each case, metformin acts as a major metabolism disruptor in cancer cells, induces dysregulation in energetic metabolism and protein synthesis, and activates autophagy and apoptosis processes.

## Metformin and melanoma

Metformin can induce cancer cell death by different mechanisms. However, what is known about metformin in melanoma cells?

As previously described, melanoma is the most aggressive form of skin cancer, and currently, efficient treatments have not been developed for most patients. The discovery of new treatments for this cancer appears to be essential. In this context, different laboratories, including ours, have shown that metformin or phenformin (another biguanide compound) can inhibit melanoma cell proliferation (33, 71–73).

**Figure 2:**
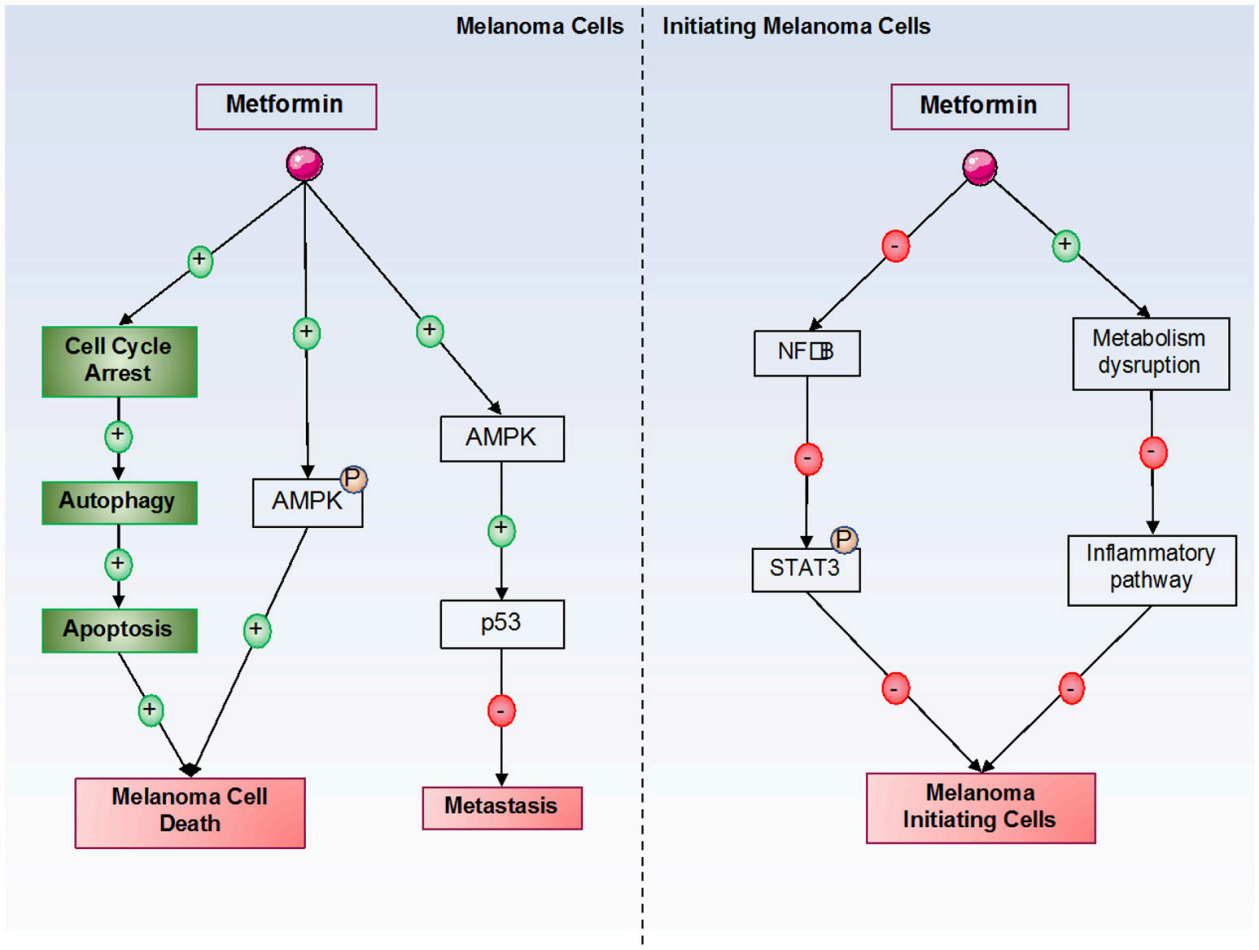
Metformin's effects on melanoma cells. Metformin induces melanoma cell death by both AMPK-dependent and -independent pathways. By an unknown mechanism, metformin induces cell cycle arrest in melanoma cells, which is responsible for the activation of autophagy, and in turn, for the activation of apoptosis, leading to melanoma cell death. In initiating melanoma cells, metformin decreased cell transformation and proliferation by inhibiting the NF-κB pathway and the inflammatory pathway.

As previously discussed, metformin can inhibit cancer cell proliferation and induce cancer cell death by many different mechanisms. In melanoma cells, it has been shown that metformin induces cell cycle arrest in melanoma cells in the G0-G1 phase after 24 h of treatment at 10 mM. However, the molecular mechanism of this cell cycle arrest has not been identified in melanoma cells. Furthermore, our laboratory has also shown that this cell cycle arrest is responsible for autophagy (at 72 h) and apoptosis (at 96 h) induction by metformin in melanoma cells (71, 74). In this model, we also showed that inhibition of AMPK (by siRNA) induces a partial restoration of melanoma cell viability under metformin treatment, suggesting that AMPK plays a partial role in metformin-induced melanoma cell death. This finding also suggests that another AMPK-independent mechanism is implicated in metformin-induced melanoma cell death (Figure 2). Interestingly, in xenograft mouse models, metformin decreases the tumoral volume of melanoma cells. In addition, no cellular death has been observed in normal human cells, such as melanocytes, even if endogenous AMPK is expressed. Similar results have also been observed by other laboratories (75, 76). In another study, metformin induced autophagy activation in melanoma cells by inhibiting a new potential therapeutic target, tribbles pseudokinase 3 (TRIB3) (33). In this study, the authors showed that metformin attenuated melanoma growth and metastasis by reducing TRIB3 expression in non-diabetic and diabetic mouse models.

Interestingly, a recent study showed that metformin can directly act not only on melanoma cells to induce cell death but also on the tumor microenvironment, particularly in the context of an immune response (77). This study showed that metformin activated both autophagy and apoptosis in melanoma cancer cells *in vitro* and confirmed the results *in vivo* in mouse models challenged with B16 murine melanoma cells. The results showed that metformin activity on melanoma cells was partly due to the immune system and that the antitumor activity of metformin was lost on immunodeficient (NSG) mice. This group also showed that metformin interaction with the immune system was principally associated with T cells (77). As described in the introduction, the immune system is very important in melanoma therapies, and current immunotherapies show very interesting objective responses, but they occur in very few patients. This study showed the interactions between the immune system and metformin; thus, it will be interesting to test a combination of metformin treatment and immunotherapies, such as anti-PD1, to increase the effects of immunotherapies in melanoma cells.

Another study from our laboratory showed that metformin inhibited the proliferation of melanoma cells (78). Indeed, in this study, we showed *in vitro* that metformin modulates the expression of different proteins, such as Slug, Snail, and matrix metalloproteinases 2 and 9 (MMP2 and 9), the latter two of which are implicated in the epithelial-mesenchymal transition *via* an AMPK- and p53-dependent mechanism. However, even if metformin can inhibit cell invasion, it has no effect on the migration ability of melanoma cells. Furthermore, *in vivo*, we showed that metformin inhibits melanoma metastasis development in the lung. In general, a study on UV-induced skin cancers showed that metformin, by an AMPK-dependent pathway, inhibited tumorigenesis in skin cells (79). Indeed, the authors showed that AMPK was inhibited by UVB irradiation, demonstrating its important role as a tumor suppressor in skin cancers. This inhibition enhanced decrease of the DNA damage response pathway *via* reduction of xeroderma pigmentosum C (XPC). However, under stimulation with AICAR and metformin, the DNA damage response pathway was reactivated, decreasing in cancer cell development. Therefore, the authors showed the importance of AMPK activation by different treatments, such as metformin, in decreasing cancer cell development and proliferation induced by UVB, such as in melanoma development.

Interestingly, melanoma is one of cancer that is the most dependent on and impacted by metabolism (80). Melanoma is a cancer that requires glycolytic metabolism, which is mediated by mitochondrial activity (81). Moreover, resistance to BRAF inhibitor cells have shown increased oxidative metabolism and mitochondrial dependence for cell survival (82). Therefore, in both sensitive and resistant to BRAF inhibitor melanoma cells, mitochondria, and metabolism appear to be essential, and a drug, such as metformin or another biguanide, that alters this metabolism could be an interesting prospect for new melanoma treatments. This information suggests that testing a drug such as metformin, which disrupts metabolism, in combination with other therapies, such as targeted therapies (BRAF inhibitors) or immunotherapies (anti-PD1) in melanoma cells, could increase objective responses and inhibit primary or acquired resistance to these treatments.

Studies have examined the effects of metformin in combination with BRAF inhibitors, such as vemurafenib (Zelboraf). Indeed, many groups have used combination treatments with BRAF inhibitors to inhibit resistance in melanoma cells. The combination of vemurafenib (BRAF inhibitor) and metformin showed encouraging results with synergistic effects for inducing melanoma cell death (76). Indeed, *in vitro* experiments show synergistic antiproliferative effects, particularly in BRAFV600E mutant cell lines. In other studies, metformin increased the toxicity of cisplatin, a chemotherapeutic drug, in melanoma cells (83, 84).

These results seem interesting, but further study is needed. Indeed, in certain studies, the combination of metformin and a BRAF inhibitor stimulated the proliferation of melanoma cells mutated by NRAS (76). It will be interesting to observe the metabolic characteristics of melanoma cells after a treatment combination of metformin and BRAF inhibitors to better understand active mechanisms.

Furthermore, metformin effects were analyzed in combination with immunotherapies (anti-CTLA4, anti-PD-1 or anti-PD-L1). As previously stated, immunotherapies have been developed for melanoma treatment for a few years. These therapies, which tend to reactivate the patient's immune system, show very efficient and durable responses, but they are effective in only 15–30% of patients. Therefore, we can imagine that combinations with another molecule, such as metformin or another biguanide can increase the objective responses obtained with immunotherapies and decrease resistance to these treatments. Interestingly, a recent study showed that phenformin, another biguanide, potentiated the effect of immunotherapy (85). In this study, the authors showed that phenformin induced the production of reactive oxygen species in granulocytic myeloid-derived suppressor cells, which increased the effect of immunotherapies on melanoma cells. Indeed, in combination with anti-PD-1, phenformin enhanced melanoma inhibition in a BRAF V600E/PTEN-null melanoma mouse model. In these mice, CD8+ lymphocytes were activated, which increased melanoma cell death. In addition, results from the study by Scharping et al. suggested that tumor hypoxia plays a role as a barrier against immunotherapy and that metformin, which can reduce intratumoral hypoxia, can improve immunotherapy efficacy against melanoma cells (86). Taken together, these results suggest that biguanides, such as metformin, could be used in combination with targeted therapies against BRAF or with immunotherapies to synergize treatment effects on melanoma cells.

Finally, certain studies have examined metformin's effects on melanoma initiating cells (MIC). Indeed, melanoma is a heterogenic tumor, and some studies including ours have suggested that MIC could be responsible for the metastatic potential of melanoma, which could be implicated in resistance to BRAF inhibitor therapies (87, 88). These MIC constitute a chemoresistant cell population that expresses specific markers. However, independently of MIC numbers, characteristics, or mechanisms that regulate the transition between MIC and proliferative cells, it is clear that melanoma cell populations with different tumorigenic potentials exist (89). A study has shown that STAT3 (Signal Transducer and Activator of Transcription 3) pathway activation is necessary to acquire “MIC properties” (90). Furthermore, a recent study showed that a combination of stattic and metformin decreased brain tumor initiating cells by STAT3-dependent mechanisms (91). Interestingly, metformin blocks the inflammatory pathway responsible for stem cell transformation and growth due to cellular metabolism disruption (84). Finally, our laboratory has shown that metformin can decrease MIC populations (unpublished results) (Figure 2).

## Clinical trials of metformin on melanoma treatment

In this context, 304 clinical trials have been or are currently being performed to test the effects of metformin on different cancer types (ClinicalTrials.gov).

In our laboratory, thanks to the *in vitro* and *in vivo* results obtained from metformin treatment against melanoma, we developed a phase II clinical trial that was performed in the dermatology department at the University Hospital Centre in Nice. This clinical trial allowed us to determine the efficacy of metformin treatment on metastatic melanoma patients (92). In this study, patients were experiencing therapeutic failure to chemotherapies and BRAF inhibitors and were not eligible for or not responsive to immunotherapy treatment. The study was performed on 17 patients with ages ranging from 49 to 88 years (mean of 74 years). Metformin was prescribed at 1,000 mg three times daily. After 4 months, 11 patients showed melanoma progression, 3 patients were deceased due to the disease and 2 patients had to stop treatment. After 6 months, the only patient still being treated with metformin did not show a significant response. These results were not very encouraging; it could have been concluded that metformin treatment at similar doses to those in type 2 diabetes did not induce significant efficacy in this population of metastatic melanoma patients, independently of mutational status. However, the poor efficacy of metformin treatment could be linked to different barriers in this study. Indeed, treated patients were in total therapeutic failure and had notable progression of melanoma disease. Therefore, it will be interesting to test metformin or new biguanide-derived compounds with better efficacy at an early stage of disease progression.

Currently, other clinical trials are still in progress, and their results will be truly important for understanding metformin treatments against melanoma and continuing their use. Another phase I/II clinical trial is currently being performed in the United States in Louisville, and metformin's treatment effects in combination with vemurafenib, a BRAF inhibitor, is being evaluated in 55 patients with BRAF-mutated melanoma (ClinicalTrials.gov, NCT01638676). At the University of Louisville, another clinical trial evaluating the combination of dabrafenib/or trametinib and metformin is being evaluated. This phase I/II clinical trial started in 2014 on 53 participants, and no results have been published (ClinicalTrials.gov, NCT02143050). Other clinical trials are currently in progress to evaluate the effects of metformin in combination with different treatments; a study of metformin combined with pembrolizumab (immunotherapy) is being conducted in Pittsburgh (ClinicalTrials.gov, NCT03311308), and a study on metformin combined with dacarbazine (chemotherapy) is being conducted in Petersburg (ClinicalTrials.gov, NCT02190838).

In conclusion, metformin, and more generally biguanides, seem to be good candidates for the development of new therapies against melanoma. Their impact on metabolism and the activation of cell death mechanisms in melanoma cells could be promising in melanoma treatment. Furthermore, studies in which metformin, or other biguanides, is combined with current therapies show a synergistic response in melanoma cells, and therefore, their results could be interesting for the development of new therapy combinations against melanoma and even other cancer cell types.

## Author contributions

EJ wrote the first draft and made the figures of the review. SR oversaw the work and made the corrections and the final modifications.

### Conflict of interest statement

The authors declare that the research was conducted in the absence of any commercial or financial relationships that could be construed as a potential conflict of interest.
